# Recent progress in pancreatic islet cell therapy

**DOI:** 10.1186/s41232-020-00152-5

**Published:** 2021-01-05

**Authors:** Erinn Zixuan Sim, Nobuaki Shiraki, Shoen Kume

**Affiliations:** School of Life Science and Technology, Tokyo Institute of Technology, 4259-B-25 Nagatsuta-cho, Midori-ku, Yokohama, Kanagawa 226-8501 Japan

**Keywords:** Pluripotent stem cells, Directed differentiation, Pancreatic islets, Transplantation, Disease modeling

## Abstract

Human pluripotent stem cells (PSCs), including human embryonic stem cells and induced pluripotent stem cells, are promising cell sources in regenerating pancreatic islets through in vitro directed differentiation. Recent progress in this research field has made it possible to generate glucose-responsive pancreatic islet cells from PSCs. Single-cell RNA sequencing techniques have been applied to analyze PSC-derived endocrine beta-cells, which are then compared with human islets. This has led to the identification of novel signaling pathways and molecules involved in lineage commitment during pancreatic differentiation and maturation processes. Single-cell transcriptomics are also used to construct a detailed map of in vivo endocrine differentiation of developing mouse embryos to study pancreatic islet development. Mimicking those occurring in vivo, it was reported that differentiating PSCs can generate similar islet cell structures, while metabolomics analysis highlighted key components involved in PSC-derived pancreatic islet cell function, providing information for the improvement of in vitro pancreatic maturation procedures. In addition, cell transplantation into diabetic animal models, together with the cell delivery system, is studied to ensure the therapeutic potentials of PSC-derived pancreatic islet cells. Combined with gene-editing technology, the engineered mutation-corrected PSC lines originated from diabetes patients could be differentiated into functional pancreatic islet cells, suggesting possible autologous cell therapy in the future. These PSC-derived pancreatic islet cells are a potential tool for studies of disease modeling and drug testing. Herein, we outlined the directed differentiation procedures of PSC-derived pancreatic islet cells, novel findings through transcriptome and metabolome studies, and recent progress in disease modeling.

## Background

Pancreatic islet cell therapy is a promising treatment for diabetes mellitus; however, it is hampered by the persistent shortage of donor islets [1]. Human pluripotent stem cells (PSCs), which possess the ability of indefinite proliferation and differentiation capacity into all cell types, are a potential source in generating pancreatic islets for replacement therapy. Upon enormous progress in this research field made in the past decade, directed differentiation of insulin-producing pancreatic islet cells using human embryonic stem cells [2] and induced pluripotent stem cells [3], has become feasible. Phase I/II clinical trials were launched in 2014 and 2017 using hESC-derived pancreatic progenitors (NCT02239354, NCT03163511). In this review, we discuss the progress of in vitro pancreatic differentiation procedures, the analysis of the PSC-derived pancreatic islet cellular identity, cell transplantation, and applications of differentiated PSCs in disease modeling.

### Growth factors and small molecules that facilitate in vitro pancreatic differentiation

Islets of Langerhans, commonly referred to as pancreatic islets, are derived from the definitive endoderm. The endoderm then folds to form the primitive gut tube, followed by region specification and organ-specific bud formation, subsequently giving rise to various respiratory, digestive organs, including the dorsal and ventral pancreas. Pancreatic buds, where the pancreatic progenitors originate, are marked by pancreatic and duodenal homeobox 1 (PDX1), a gene required for pancreatic growth. Developmental processes continue with an expansion of these pancreatic progenitors followed by fate divergence into exocrine and endocrine progenitors. The morphogenetic processes and expression profiles of key lineage-specific genes that contributed to pancreatic development in vertebrates were studied, and this knowledge was applied to in vitro directed differentiation of pancreatic islet cells from PSCs [4–6].

**Fig. 1:**
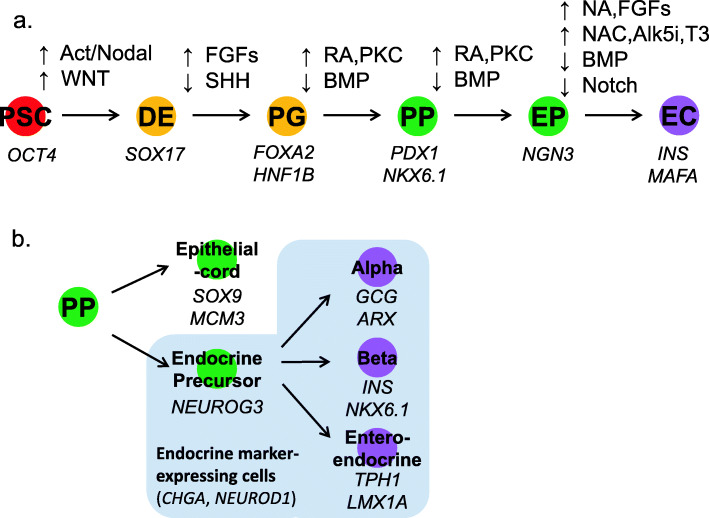
Directed differentiation of PSCs into pancreatic beta cells. **a** The schematic diagram of directed differentiation into pancreatic beta cells using PSCs. PSCs are directed through 5 stages; each stage utilizes the inhibition (↓) or activation (↑) of various signaling pathways. Genes specifically expressed at each stage are shown. **b** Genes expressed in various cell groups revealed from scRNA-seq analysis. PSCs pluripotent stem cells, DE definitive endoderm, PG primitive gut tube, PP pancreatic progenitor, EP endocrine progenitor, EC endocrine cell, SHH sonic hedgehog, FGFs fibroblast growth factors, BMP bone morphogenic protein, RA retinoic acid, PKC protein kinase C, NA Nicotinamide, NAC *N*-acetyl-cysteine, Alk5i transforming growth factor-beta receptor inhibitor, T3 thyroid hormone

Stepwise pancreatic differentiation protocols from PSCs are established by mimicking the in vivo pancreatic development processes. PSCs are sequentially differentiated into definitive endoderm (DE), primitive gut tube (PG), pancreatic progenitor (PP), endocrine progenitor (EP), then into hormone-expressing endocrine cells (EC). The in vitro differentiation procedures utilize the inhibition or activation of WNT, transforming growth factor-β (TGF-β) superfamily signaling pathways, such as Activin, Nodal, and Bone Morphogenic Protein (BMP), retinoic acid (RA), and protein kinase C (PKC) signaling (Fig. 1a). Combined treatment of the above growth factors and small molecules were adopted for the directed differentiation of pancreatic endocrine beta cells (Table 1).

**Table 1:** A list of growth factors and small molecules adopted in the in vitro differentiation procedures

Stages	Growth factors/small molecules	Signaling pathways	Functions	References	
				Rodent in vivo	Human in vitro
**DE**	Activin A	TGF-β superfamily member	Induce DE differentiation	[7, 8]	[9, 10]
	Wnt3a	TGF-β superfamily	Induce mesendoderm differentiation	[11]	[12]
	CHIR99021	GSK3β inhibitor (WNT)	Promote SOX17 expression better than Wnt3a	**–**	[13]
**PG**	FGF10	FGF signaling	Induce PG differentiation	[14, 15]	[12]
	KAAD-cyclopamine	SHH inhibitor	Enhance DE differentiation	[6, 16]	[12]
	KGF, FGF7	FGF signaling	Enhance PG and differentiation to the pancreatic lineage	[4, 17]	[18–20]
**PP, EP**	RA	RA signaling	Promote PDX1 expression	[4–6]	[10]
	SANT1	SHH inhibitor	Enhance DE differentiation	[6, 16]	[18]
	Noggin, LDN193189	BMP inhibitors	Enhance differentiation to the pancreatic lineage	[4]	[18–22]
	ILV, PDBu, TPB	PKC activator	Promote PDX1 or NKX6.1 expression	**–**	[18, 19, 23]
	EGF	EGF signaling	Enhance PP proliferation	[4, 24]	[22, 25, 26]
	Sodium cromoglicate	BMP inhibitor	Promote NGN3 expression	**–**	[27]
**EC**	Alk5i	TGF-β receptor inhibitor	Induce differentiation to the endocrine lineage from PP	[4, 6]	[18]
	Nicotinamide	Amide form of Vitamin B3	Induce EC differentiation	**–**	[10, 21, 22, 28]
	N-acetyl cysteine	Antioxidant	Promote MAFA nuclear localization	[29]	[20]
	T3	Thyroid hormone	Promote co-expression of NKX6.1 and INS	[30]	[19, 20]
	γ-secretase inhibitor XX	Notch inhibitor	Enhance differentiation to the endocrine lineage	[31, 32]	[19, 30]
	H1152	ROCK inhibitor	Promote INS expression	**–**	[33]
	R428	AXL inhibitor	Promote MAFA production	**–**	[20]

*DE* definitive endoderm; *PG* primitive gut tube; *PP* pancreatic progenitor; *EP* endocrine progenitor; *EC* endocrine cell; *GSK3β* glycogen synthase kinase 3 beta; *FGF* fibroblast growth factor; *KAAD-cyclopamine* 3-keto-*N*-(aminoethylaminocaproyl-dihydrocinnamoyl) cyclopamine; *SHH* sonic hedgehog; *KGF* keratinocyte growth factor; *RA* retinoic acid; *BMP* bone morphogenic protein; *ILV* Indolactom V; *PDBu* Phorbol 12, 13-Dibutyrate; *TPB* (2S,5S)-(E,E)-8-(5-(4-(trifluoromethyl)phenyl)-2,4-pentadienoylamino) benzolactam; *PKC* protein kinase C; *EGF* epidermal growth factor; *Alk5i* transforming growth factor beta receptor inhibitor; *T3* thyroid hormone

DE differentiation from PSCs involves the activation of Activin/Nodal and WNT signaling. Inactivation of *Wnt3* during mice embryogenesis resulted in defective primitive streak formation [11]. The endoderm failed to form in the *Nodal-Smad2* mutant mice [7]. This knowledge was applied for DE induction from PSCs. Activin is used since it binds to and activates the endogenous Nodal receptor [8]. D’Amour et al. successfully directed PSCs into DE cells under low serum and high Activin A concentration [9]. They later improved the DE differentiation efficacy by adding a WNT protein, Wnt3a, on the first day of Activin A treatment [12]. In consideration of differentiation efficiency, stability, and cost, Kunisada et al. substituted Wnt3a with CHIR99021, a highly selective glycogen synthase kinase 3 beta (GSK3β) inhibitor. Treatment of CHIR99021, together with Activin A, induced a higher percentage of DE cells compared to Wnt3a and Activin A [13].

PG formed after the establishment of DE, coincident with a transition from a two-dimensional cell sheet into a three-dimensional tube-like structure [6]. Fibroblast growth factors (FGFs) and sonic hedgehog (SHH) signaling pathways take part in this event. In chick embryos, *Shh* is expressed along the gut tube but is absent in the pancreatic endoderm. The removal of notochord results in an induction of *Shh* expression in the pancreas and a loss of pancreatic gene expression, suggesting that the repression of *Shh* is permissive for early pancreatic development [6, 16]. *Fgf10* is expressed in the mesenchyme adjacent to the early dorsal and ventral pancreatic epithelial buds. Upon deletion of *Fgf10* in mouse embryos, subsequent growth, differentiation, and branching morphogenesis of the pancreatic epithelium are arrested [14]. On the other hand, *Fgf10*-expressing transgenic mice exhibited cell proliferation of the pancreatic epithelium [15]. PSC-derived DE cells cultured with SHH signaling inhibitor, 3-keto-*N*-(aminoethyl-aminocaproyl-dihydrocinnamoyl) cyclopamine (KAAD-cyclopamine), and FGF10 resulted in PG induction [12].

Signaling pathways involving RA, PKC, BMP signaling, and others were reported to be potent in inducing PP and EP in vitro. Jiang et al. reported that the addition of all-trans retinoic acid contributed to the increased PDX1-positive cell population [10]. Exogenous addition of epidermal growth factor (EGF) was shown to increase the size of E13.5 rat pancreatic buds under 7 days of ex vivo culture [24] and was used to facilitate the expansion of the human PSC-derived PDX1-positive PPs under in vitro culture [25]. A combination of Indolactom V (ILV), a PKC agonist identified from a chemical compound screening, and FGF10 effectively increased the expressions of pancreatic lineage markers, while decreasing the expression of non-pancreatic tissue markers in three PSC lines [23]. Another PKC activator, (2S,5S)-(E,E)-8-(5-(4- (Trifluoromethyl)phenyl)-2,4-pentadienoylamino) benzolactam (TPB), used with Noggin (a BMP signaling inhibitor) and Alk5 inhibitor (a TGF-β receptor inhibitor, Alk5i), also elevated the *PDX1* expression level [18]. Shahjalal et al. reported that a high dose of Noggin treatment significantly increased *PDX1* expressing cells, suggesting that inhibition of BMP signaling potentiates pancreatic differentiation and suppresses hepatic and intestinal differentiation [21]. A high percentage of cells co-expressing PDX1 and another important marker for endocrine development [4, 6], NK6 homeobox 1 (NKX6.1), consequently enhances the efficiency of in vitro differentiation into EP cells. A short duration of treatment with RA, FGF10, BMP, and SHH signaling pathways inhibitors modulates the efficiency of differentiation into PPs, contributing to a higher percentage PDX1 and NKX6.1 co-expressing cells [22]. Even with the omission of BMP inhibitors, treatment with RA followed by combined treatment with EGF and keratinocyte growth factor (KGF) efficiently increased the number of both PDX1-expressing cells and subsequent PDX1/NKX6.1+ cells [26]. KGF increases beta-cell population through activation of the protein kinase B/Akt signaling pathway [17]. Neurogenin 3 (*NEUROG3*) expression defines the pancreatic EP cells, which later differentiate into ECs [34]. Continuous Noggin treatment during PP and EP stage can induce in vitro differentiation into *NEUROG3*-expressing cells [21]. A small molecule, sodium cromoglicate, identified from a chemical screening, was reported to facilitate differentiation into PDX1-expressing cell population derived from PSCs. Sodium cromoglicate treatment subsequently doubled the percentage of NEUROG3-positive cells compared to untreated controls [27].

During the differentiation of EP cells into ECs, various growth factors and small molecules are used to increase INSULIN-(INS)-positive EC cells. Treatment of the human fetal pancreatic islet cells with Nicotinamide (the amide form of vitamin B3) resulted in increased DNA content, thereby suggesting the expansion of the pancreatic progenitor cells, as DNA synthesis was stimulated only in the nonhormone-expressing cells [28]. Nicotinamide and FGFs were used to enhance INS-expressing EC differentiation from EP cells [10, 25]. Inhibition of the BMP signaling pathway was essential for generating *INS*-expressing cells [35]. Thyroid hormone (T3) and γ-secretase inhibitor XX (a Notch pathway inhibitor) are also being used to promote EC differentiation from PSCs [19]. T3 promotes pancreatic beta-cell maturation in rats [30]. In mice, misexpression of activated Notch in Pdx1-expressing progenitor cells prevents differentiation into endocrine lineage [31]. In contrast, reduced Notch signaling leads to increased expression of *Neurog3* that plays a key role in determining the endocrine fate [32]. From a high content chemical screen attempting to identify chemicals that facilitated INS-expression in cells, a ROCK inhibitor, H1152, was identified to increase the percentage of INS-expressing cells from PSCs [33]. Monoamine (dopamine, in particular) acts as a negative regulator that arrests the differentiation of PSCs at the PP stage. Reserpine and tetrabenazine, vesicular monoamine transporter 2 (VMAT2) inhibitors, are hit compounds identified in another high-throughput chemical screening. Adding these compounds to PSC culture decreased cellular dopamine content and potentiated late-stage differentiation into INS-expressing cells [36]. Rezania et al. focused on *MAF bZIP transcription factor A* (*MAFA*), a critical beta-cell maturation marker gene [20]. They proposed that combined exposure of R428 (a small-molecule inhibitor of tyrosine kinase receptor AXL), a high dose of *N*-acetyl cysteine (an antioxidant; increased antioxidants in mice beta-cells resulted in preservation of nuclear MAFA [29]), Alk5i II (TGF-β receptor), and T3 treatment resulted in increasing *MAFA* transcription levels.

Among all the above growth factors and small molecules used during pancreatic differentiation, the combination, dosage, and exposure time of these components are important to obtain functional hormonal-expressing ECs. Table 2 showed a list of components used in several reports which successfully generated functional ECs using PSCs. With systematic testing of concentration and exposure time of numerous factors, Pagliuca et al. developed a protocol with the combination of various factors affecting pathways, including WNT and Activin/Nodal (DE differentiation), SHH, EGF, TGF-β, and RA (PG, PP, and EP differentiation), T3 and γ-secretase inhibitor XX (a Notch pathway inhibitor). As a result, they successfully generated PSC-derived pancreatic islet cells that expressed not only mature beta-cell markers but were also capable of insulin packaging and improved glucose-stimulated insulin secretion [19]. With a combination of R428, a high dose of *N*-acetyl cysteine, Alk5i II, and T3 treatment, Rezania et al. successfully increased the *MAFA* transcription levels in ECs. The pancreatic islet cells generated from PSCs expressed the key markers of mature pancreatic beta-cells, including *MAFA*. The PSC-derived pancreatic beta-cells displayed improved glucose-stimulated insulin secretion (GSIS) [20]. Velazco-Cruz et al. reported that the removal of Alk5i and T3 at the last stage of induction is crucial for generating functional beta-cells. The PSC-derived ECs exhibited increased insulin secretion and glucose responsiveness, with a dynamic first- and second-phase insulin release at a level similar to that observed in human islets [38].

**Table 2:** A list of growth factors or chemicals used for generating functional PSC-derived ECs

References		Pagliuca et al., 2014 [19] Millman et al., 2016 [37]	Rezania et al., 2014 [20]	Velazco et al., 2019 [38]
**Stages**	**DE**	Activin A	GDF8	Activin A
		CHIR99021	GSK3β inh	CHIR99021
	**PG**	KGF	FGF7	KGF
			Vitamin C	LDN193189
	**PP, EP**	LDN193189	Vitamin C	KGF
		KGF	RA	SANT-1
		SANT-1	SANT-1	PDBu
		PDBu	TPB	RA
		RA	LDN	Y27632
		Y27632	Alk5 inh	Activin A
		Activin A	T3	
			LDN193189	
	**EC**	SANT-1	Alk5 inh	SANT-1
		RA	T3	RA
		GSXX inh	LDN	GSXX inh
		Alk5 inh	GSXX inh	Alk5 inh
		T3	NAC	T3
		Betacellulin	AXL inh	Betacellulin
		Heparin		

Present research progress has shown that regeneration of pancreatic islets using PSCs is not an unreachable goal. There are remaining tasks in investigating the similarity and differences between PSC-derived ECs and human islets, not only with regard to the cell culture microenvironment and molecular characteristics, as well as long-term maintenance of the function of the PSC-derived ECs. Recent progress regarding the cell culture microenvironment, together with transcriptome and metabolome profiles of PSC-derived ECs resulted from the differentiation procedure above, will be discussed in the later sections.

### Cell culture microenvironment in differentiating PSCs

Apart from the optimization of medium components, several studies highlighted that cell culture system, such as adherent culture, culture in suspension, or reaggregation culture, during differentiation could affect pancreatic differentiation. Directed differentiation was initially done using adherent culture systems. In an attempt to overcome surface-area constraints, achieve manufacturing-scale culture, and prepare enough cells for transplantation, suspension procedures were proposed [39, 40]. Further experiments showed that cell aggregation promoted the generation of PDX1/NKX6.1-positive cell populations derived from PSCs [41]. Controlling the size of the cell clusters at the final stage of differentiation [38] or isolating and reaggregating ECs into islet-sized clusters [42] resulted in cells of higher quality that exhibited dynamic insulin secretion. Results on the single-cell analysis of PPs derived from PSCs revealed that cell confinement is a prerequisite for endocrine specification [43], emphasizing the necessity of cell clustering in islet formation. Late-stage differentiation has also been performed in an air-liquid interface culture environment, mimicking an environment that may modulate beta-cell differentiation [20].

The state of the cytoskeleton was reported to affect the expression levels of transcription factors for endocrine differentiation. PSCs cultured under adherent conditions were reported to yield a decreased differentiation efficiency into ECs. This issue was overcome by treatment with a cytoskeletal depolymerizer latrunculin A. Under adherent cultures of three PSC cell lines, latrunculin A treatment yielded islet cells exhibiting key markers of mature pancreatic cells and dynamic insulin secretion with first- and second-phase [44]. A detailed map of endocrine differentiation was constructed using a single-cell transcriptome of the developing mouse pancreatic islets. These results showed that islet formation does not involve migration and aggregation of multiple individual cells. Instead, the islets form as budding peninsulas, or more commonly known as endocrine budding. Early differentiating alpha-cells appear at the tip of the peninsula, and the later differentiating beta-cells form in the rear. Under suspension culture conditions and optimized pancreatic differentiation procedures of the PSCs, bud-like protrusions appeared from the bulk of the spheroids (cell clusters of beta-like cells). Flow cytometry analysis revealed that the cells in the buds expressed a higher percentage of Chromogranin A (CHGA), INS, and Glucagon (GCG) compared to the cells in the bulks. The results suggest that PSCs can be induced in vitro to generate hormone-secreting cells within a peninsula-like structure similar to that observed during developmental processes in the mouse [45]. Overall, studying the connection between cell culture microenvironment and endocrine differentiation might lead to a more efficient regeneration of pancreatic islets in vitro.

### Single-cell RNA sequencing revealed heterogeneous cell population and lineage bifurcations in PSC-derived ECs

Given that significant progress has been made in directed differentiation of pancreatic islet cells from PSCs, recent investigations utilized transcriptome sequencing and metabolome analysis of the differentiating PSCs to characterize their cellular identity. Compared to the bulk RNA sequencing result of PSC-derived INS-expressing cells before aggregation, reaggregated PSC-derived INS-expressing cell clusters showed a higher degree of correlation with beta-cells isolated from adult human islets [42]. From single-cell RNA sequencing (scRNA-seq) analysis, Veres et al. reported a heterogeneous cell population in PSC-derived islet cells, showing four major cell populations. They are (1) beta-cells that express *INS*, *NKX6.1*, and other beta-cell markers; (2) polyhormonal alpha-like cells that express *GCG*, Aristaless-related homeobox (*ARX*), and *INS*; (3) enterochromaffin cells and endocrine cell types expressing *CHGA*, Tryptophan hydroxylase 1 (*TPH1*), LIM homeobox transcription factor 1 alpha (*LMX1A*), and Solute carrier family 18 member A1 (*SLC18A1*); and (4) SRY-Box Transcription Factor 9-(*SOX9*)-expressing non-ECs (a pancreatic duct marker of exocrine cells). A surface marker of PSC-derived beta-cells, CD49 antigen-like family member A (CD49a), was identified, and pure PSC-derived beta-cell clusters were generated [46]. In vitro directed differentiation into pancreatic islet cells was performed in a stepwise manner as shown in Fig. 1a. Petersen et al. and Sharon et al. showed lineage bifurcations or two waves of endocrine differentiation after the PP stage, analyzed by scRNA-seq of PSC-derived ECs. Petersen et al. found lineage convergence among the EP cells, focusing on the expression of motor neuron and pancreas homeobox (*MNX*). *MNX*-positive cells will eventually express *NKX6.1* and E-twenty-six (ETS) transcription factor, Fifth Ewing variant (*FEV*). *FEV* expression precedes LIM homeobox 1 (*ISL1*) in EP cells that mark cells on the beta-cell pathway. *MNX-*negative cells in turn give rise to the *FEV-*negative *ISL1-*positive cell population which further progress into *ARX*- and *INS*-positive cells, attesting to the polyhormonal characteristics of the cells [47]. Sharon et al. reported that homogeneous differentiation only occurs in the first three stages (DE, PG, and PP). From EP onwards, the clusters contained heterogeneous populations, including epithelial-cord markers and endocrine markers-expressing cells (endocrine precursors, alpha-cell markers-, beta-cell markers-expressing cells, and enteroendocrine cells) (Fig. 1b). There were two waves of endocrine differentiation, in which alpha-cells started to form in the first wave. The second wave gives rise to beta-cells and non-pancreatic ECs. Also, they reported that WNT inhibition plays a role in endocrine differentiation. Treatment of WNT-tankyrase inhibitor, IWR1-endo increased the percentage of the endocrine marker, CHGA, and PP markers, PDX1/NKX6.1-positive cell populations [48].

In a recent report, Helman et al. performed a metabolic analysis of PSC-derived beta-cells, focusing specifically on their glucose responsiveness. The authors investigated the possibility that sensing environmental nutrients by mechanistic target of rapamycin complex 1 (mTORC1) contributes to islet cell maturation. They found that the mTORC1 pathway was strongly activated in fetal beta-cells, while adult human beta-cells showed low mTORC1 activity. The activated mTORC1 was responsive to glucose and amino acids. By culturing PSC-derived beta-cells under an amino acid-rich, but not glucose-rich medium, activation of the mTORC1 signaling pathway was observed, evidencing their similarity with fetal beta-cells. Inhibition of the mTORC1 signaling with an mTOR inhibitor, Torin1, improved glucose-responsive insulin secretion of PSC-derived beta-cells [49]. The finding suggests a transition of nutrient-sensitivity in the mTORC1 pathway triggers glucose-responsive insulin secretion. Utilizing this mTORC1 signaling pathway led to the improvement in generating functional insulin secreting-beta-cells derived from PSCs. Davis et al. investigated glucose metabolism and sensing in PSC-derived beta-cells and found that a bottleneck in glycolysis inhibits glucose response. They reported that the glucose sensing and regulation of insulin secretion had not achieved the level of those observed with human islets in vitro. During glucose challenges, glucose-induced anaplerotic flux was observed in the human islets through pyruvate carboxylase (a mitochondrial enzyme that catalyzes the carboxylation of pyruvate to oxaloacetate during glycogenesis). However, this was not observed in PSC-derived beta-cells, thereby suggesting a deficiency exists in the glycolytic pathway. They further showed that the application of metabolites from late glycolysis could rescue the PSC-derived beta-cells to acquire insulin secretion activity [50].

Functional islets derived from PSCs can be analyzed using scRNA-seq to determine novel signaling pathways and lineage commitment of PSCs during pancreatic differentiation and maturation. At the same time, metabolome studies enable us to acquire information on PSC-derived islet cell function. These approaches will provide us a clearer picture to understand the specific molecules involved and the exact timing for differentiation cues, facilitating the future development of directed differentiation to generate functional pancreatic islets from the PSCs.

### Transplantation of pancreatic islet cells derived from PSCs

As islet transplantation progressed over the past decades, these following aspects are reported to be ideal for transplant procedures: ready access to oxygen, glucose and insulin delivery, minimal procedural risk, long-term functionality and low inflammatory reaction, and monitoring of islet after transplantation. To reduce substantial islet cell loss and graft inflammation, alternative transplantation sites, cell sources, and encapsulation technologies were studied to improve islet transplantation [51, 52].

PSC-derived pancreatic beta-cells generated in vitro via directed differentiation mentioned above, as an alternative cell source, were transplanted into the immunocompromised diabetic mouse models to verify their function under in vivo environment [19, 20]. Immunocompromised mice, such as SCID-Beige, NOD SCID, or NSG mice, treated with streptozotocin (STZ) to induce diabetes, or immunodeficient NRG-Akita diabetic mice were used. Pagliuca et al. reported that fasting blood glucose levels after PSC-derived pancreatic cell transplantation reversed to normal glycemia, compared to progressively increased blood glucose levels in the control mice [19]. Rezania et al. reported a decrease in fasting blood glucose levels after two weeks of transplantation into STZ-diabetic mice. However, it was after 40 days post-transplantation that blood glucose levels significantly reduced beyond pre-STZ levels. Hyperglycemia was observed after graft removal, showing a lack of insulin secretion in the circulation post-nephrectomy [20].

**Fig. 2:**
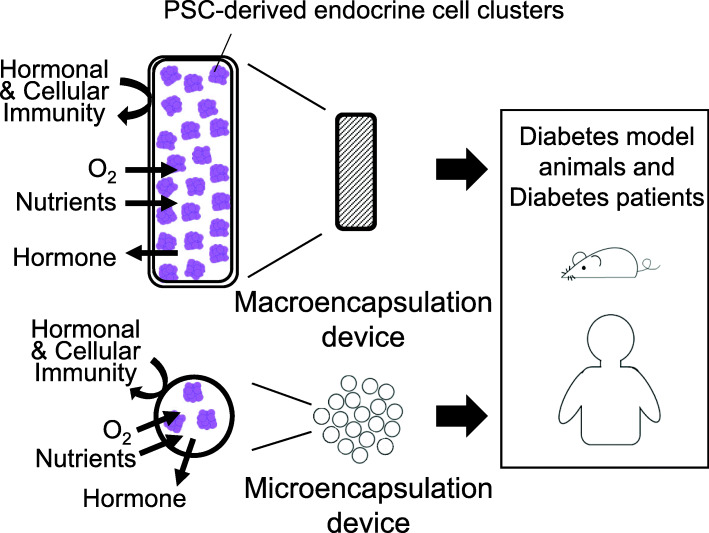
Macro- and micro-encapsulation devices for the delivery of PSC-derived islet cells. The delivery system of PSC-derived islet cells focusing on macro- and micro-encapsulation devices. Both devices can be loaded with PSC-derived endocrine cell clusters, protect the cells from immune cells, and allow penetration of oxygen, nutrients, and hormone flow

The functionality of the engrafted PSC-derived pancreatic islet cells can be confirmed on the condition that transplanted cells exhibited a high population of INS- or other mature beta-cell marker-expressing cells [19, 20, 38, 42, 44]. Further efforts are expected to establish the delivery system of PSC-derived islet cells. In 2018, Gamble et al. had reviewed a novel approach to graft implantation: the use of islet encapsulation devices [51]. Macro- and microencapsulation devices that allow penetration of oxygen and nutrients, hormone flow, and protect inner cells from humoral and cell-mediated immunity are preferable (Fig. 2). ViaCyte, Inc. (San Diego, California) developed a planar macro encapsulation device that can be loaded with functional pancreatic progenitors derived from PSCs (PEC-01) and implanted subcutaneously. The implanted cell/device combination product, VC-01, underwent Phase I/II clinical trials in 2014 (NCT02239354). ViaCyte launched a second trial (NCT03163511) using the PEC-01 cells encapsulated in a novel device, VC-02, in 2017. The novel device allows neovascularization that might improve the survival of the encapsulated cells but would require long-term immunosuppression. On the other hand, microencapsulation devices are made up of polymer-based biomaterial that encapsulates individual islet or islet clusters. Long-term glycemic correction of more than 100 days was achieved in diabetic mice engrafted with PSC-derived pancreatic islet cells encapsulated with chemically modified alginate [53]. Follow-up studies are yet to be done on investigating the efficiency and safety of long-term implantation as well as the cost for cell therapy.

### Combining gene-editing technology and directed differentiation open a new avenue to disease modeling and drug testing

PSC technology is opening the door to study specific disease mechanisms and therapies via disease modeling and drug testing as the generation of PSCs using somatic cells from patients has become possible [54]. There are successful cases of PSCs generated from somatic cells and fibroblasts of diabetic patients [55, 56]. Pancreatic islet cells generated from PSCs of type 1 diabetic patients expressed mature beta-cell markers, and responded to glucose challenge, did not show major differences with non-diabetic patients [37, 57]. Fibroblast cells from patients with single mutation-caused diabetes, maturity-onset diabetes of the young (MODY) can be reprogrammed into PSCs, expressing pluripotency markers and the ability to differentiate into cells derived from three germ layers [58, 59]. PSCs derived from MODY patients were differentiated into pancreatic cells, however, did not have significant changes in immunostaining for PDX1 and gene expression for pancreatic differentiation markers, such as *NKX6.1* and *INS* compared to control individual [60, 61].

**Fig. 3:**
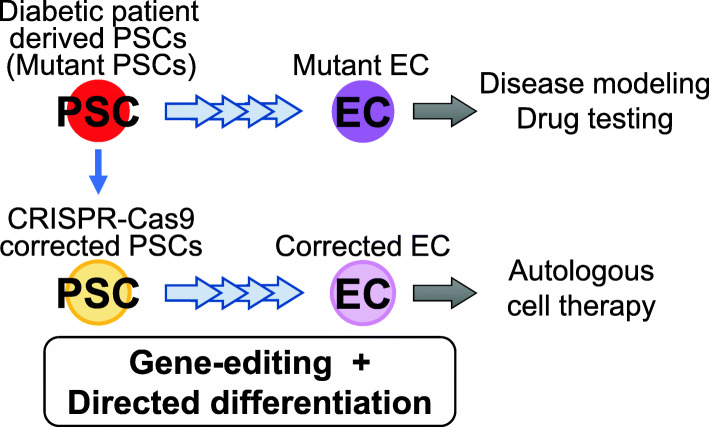
Combining gene-editing and directed differentiation for the development of novel therapeutics. Patient-derived endocrine cells can be applied in disease modeling and drug testing, while corrected PSC-derived endocrine cells can be a promising source for autologous cell therapy

Recent progress was made through optimization of the differentiation procedures and application of gene-editing technology, the CRISPR-Cas9 system, using PSCs from diabetic patients. Unlike type 1 diabetic patients, these patients carry specific gene mutations that cause diabetes [62, 63]. Balboa et al. generated PSCs from patients carrying INS mutations, used CRISPR-Cas9 to engineer mutation-corrected lines, and differentiated them to beta-like cells. Comparing the INS mutant and corrected beta-like cells, the mutant-derived beta-cells showed increased endoplasmic reticulum (ER) stress and reduced cell proliferation [62]. From the corrected PSCs derived from permanent neonatal diabetes mellitus (PNDM) patients, insulin-secreting pancreatic islet cells were generated, and upon transplantation into mice protected the mice from diabetes [64]. Maxwell et al. corrected a diabetes-causing pathogenic variant in Wolfram syndrome 1 in PSCs derived from a Wolfram syndrome patient using CRISPR-Cas9. By applying the adhesion culture system reported from the same group [44], unedited and corrected PSCs were differentiated into C-PEPTIDE (a by-product of INS formation)-positive beta-cells, co-expressing NKX6.1 and PDX1. C-PEPTIDE/NKX6.1-positive cell population was lower in the unedited compared to the corrected PSC-derived beta-cells. The gene-edited PSC-derived beta-cells displayed increased insulin secretion and higher glucose-responsiveness compared to controls. Upon transplantation, the cells successfully normalized blood glucose and reversed pre-existing diabetes in mice [63]. The result shows that directed differentiation and gene editing can be combined in the study of disease modeling, suggesting promising results for autologous cell therapy (Fig. 3). Drug testing and pathological studies of diabetes using islet cells derived from the patient’s PSCs are expected to be useful for the development of therapeutic drugs.

## Conclusions

Generation of functional pancreatic islet cells using PSCs had become possible based on the substantial progress made over the past years. Also, transplanted PSC-derived islet cells have shown the ability to normalize pre-existing hyperglycemia in mice. Follow-up research in the next decade would likely be focused more on molecular mechanisms of PSC-derived islet cells, delivery systems for transplantation, and experimental disease treatment involving the application of patient PSC-derived islet cells for therapeutic efficacy and safety considerations.

## Data Availability

Not applicable

## Abbreviations

ARX: Aristaless-related homeobox
BMP: Bone morphogenic protein
CHGA: Chromogranin A
DE: Definitive endoderm
EC: Endocrine cell
EGF: Epidermal growth factor
EP: Endocrine progenitor
FEV: Fifth Ewing Variant
FGF: Fibroblast growth factor
GCG: Glucagon
INS: Insulin
ISL1: ISL LIM homeobox 1
MAFA: MAF bZIP transcription factor A
MNX: Motor neuron and pancreas homeobox
mTORC1: Mechanistic target of rapamycin complex 1
NEUROG3: Neurogenin 3
NKX6.1: NK6 homeobox 1
PDX1: Pancreatic and duodenal homeobox 1
PG: Primitive gut tube
PKC: Protein kinase C
PP: Pancreatic progenitor
PSCs: Pluripotent stem cells
RA: Retinoic acid
scRNA-seq: Single-cell RNA sequencing
SHH: Sonic Hedgehog
TGF-β: Transforming growth factor-β
VMAT2: Vesicular monoamine transporter 2
